# Interpretable prediction of knee joint loading during tennis serves based on GNN-GRU model and layer-wise relevance propagation

**DOI:** 10.1177/09544119251361341

**Published:** 2025-10-04

**Authors:** Jianqi Pan, Zhanyi Zhou, Zixiang Gao, Diwei Chen, Fengping Li, Julien S Baker, Yaodong Gu

**Affiliations:** 1 Faculty of Sports Science, Ningbo University, Ningbo, China; 2 Faculty of Kinesiology, University of Calgary, Calgary, AB, Canada; 3 Department of Sport and Physical Education, Hong Kong Baptist University, Hong Kong, China

**Keywords:** Graph neural network, gated recurrent unit, tennis, lower limb biomechanics, explainable machine learning

## Abstract

The knee resultant joint moment is a critical indicator for assessing risk during the tennis serve. Traditional methods for obtaining this metric rely on laboratory-based equipment, limiting practical application. To address this limitation, this study proposes and validates a novel method for predicting the knee resultant joint moment method using a Graph Neural Network and Gated Recurrent Unit (GNN-GRU) model. An independent GRU model was used as a baseline for comparison. Biomechanical data were collected from 30 male tennis players (age: 20.30 ± 1.66 years, height: 176.60 ± 2.74 cm, weight: 70.80 ± 3.89 kg, BMI: 22.71 ± 1.38 kg/m^2^, training experience: 9.20 ± 2.81 years) during the performance of the tennis serve. Sagittal plane joint angles of both lower limbs were used as model inputs to predict the resultant joint moment of the supporting leg. A paired-sample t-test compared predicted and actual values. Layer-wise Relevance Propagation (LRP) was applied to quantify the contribution of individual joint angles. The GNN-GRU model demonstrated significantly better prediction performance than the standalone GRU model (*p* < 0.05). No significant differences were observed between predicted and actual values (*p* > 0.05). LRP results showed knee contribution close to 1 during the Preparation Phase (PP). In the Flight Phase (FP), ankle and hip contributions increased significantly, both approaching 1. During the Landing Phase (LP), the knee joint maintained a contribution above 0.4. This study supports the identification of potentially high-risk movements in real-world tennis training and competition and provides a reference for the early detection of knee joint injuries.

## Introduction

The tennis serve is among the most critical and technically demanding movements in this sport,^1^ requiring efficient force transmission from lower extremities and core musculature upward toward the upper limbs to enable powerful ball striking.^2^ During this process, the lower limbs are subjected to substantial mechanical loads, making them susceptible to sports injuries,^3–5^ with the knee joint being particularly vulnerable,^6^ accounting for approximately 20% of all reported cases.^7^ Several studies have identified abnormal joint moments as a high-risk factor for injury.^8–10^ Therefore, accurate estimation of knee joint moments is crucial. Resultant joint moment represents the combined influence of all internal and external forces and moments acting around a joint.^11^ It enables the assessment of overall joint function without the need for invasive measurements, and has therefore been widely applied in sports and clinical gait analysis.^12^ Changes in the resultant joint moment are closely associated with the mechanical loading of the joint. At the knee, an increased moment significantly elevates tissue stress, making it a key indicator for evaluating joint load and injury risk.^13^ Knee joint injuries are often attributed to combined mechanical loads resulting from multi-planar displacements.^14^ The resultant joint moment, generated by muscles, joint contact, and ligaments, can help identify tissues at risk of injury.^15^ In this study, it was incorporated into injury risk assessment to provide a more comprehensive evaluation of knee joint loading.

Traditionally, joint moments are calculated using forward or inverse dynamic approaches, which depend on specialized lab-based tools like force platforms and motion tracking systems.^16^ However, these measurements are difficult to implement during real tennis performances in court environments. In contrast, kinematic data are easier to collect.^17,18^ Therefore, dynamic parameters can be estimated based on kinematic data, enabling the indirect prediction of joint moments in non-laboratory settings.^19^ This study selected resultant joint moments calculated through inverse dynamics in a laboratory setting for model training and validation. Although not yet applied in real on-court scenarios, the proposed method may offer a useful reference for future estimations of joint loading using wearable sensors in field environments. Many models capable of predicting continuous time-series variables have been introduced and widely applied in the estimation of biomechanical metrics like joint moments, ground reaction forces, and pressure center.^20–24^ In the field of tennis, numerous studies have begun to utilize neural networks to identify stroke patterns,^25–27^ analyze serving motions through wearable sensors,^28^ or employ models to optimize training strategies.^29^ Whiteside et al.^25^ used support vector machines with inertial sensors to classify strokes, but their model focused only on shot type recognition without estimating biomechanical loads or offering interpretability. Li et al.^26^ applied a CNN-based video system to evaluate joint angles during volley practice, yet it lacked kinetic prediction and biomechanical insight. Other studies^28,29^ explored wearable sensing or psychological modeling but did not address interpretable joint-level kinetic estimation. Overall, prior work mainly focuses on classification or monitoring tasks, while few studies have attempted to predict joint loading in tennis using interpretable models. Among these models, Long Short-Term Memory (LSTM) networks, valued for their robustness, have become one of the most commonly adopted models in this field.^30,31^ However, traditional LSTM models have complex architectures and suffer from gradually decaying temporal correlations across time steps, making it difficult to reliably capture long-range dependencies.^32,33^ Although this limitation may be less relevant for short-duration tennis movements, LSTM models are inherently unable to capture the topological relationships between joints,^34^ which is critical for biomechanical applications. The Gated Recurrent Unit (GRU), considered a streamlined substitute for LSTM, utilizes only two gates, update and reset, while still preserving the ability to model temporal dependencies and avoiding these limitations.^35^ Meanwhile, Graph Neural Networks (GNN) offer the ability to represent structural relationships between nodes.^36^ The use of GNN in biomechanical analysis, including sports training and injury prevention, has been explored in recent investigations, which have confirmed its reliability.^37,38^ In theory, combining GNN with GRU enables the spatiotemporal modeling of graph-structured data. Existing research has shown that GNN-GRU models provide better interpretability and decision transparency than traditional LSTM models in motion prediction tasks based on joint angles.^39^ However, like all neural network models, the GNN-GRU model also faces the black-box issue, thereby hindering the ability to assess the rationality of its decisions.^40^

As a model interpretability technique, Layer-Wise Relevance Propagation (LRP) is capable of quantifying the contribution magnitude of each input variable to the model’s prediction, thereby enabling the evaluation of the plausibility of its outputs,^41^ and it has already been applied in biomechanics.^42^ Therefore, this study constructs a GNN-GRU model to predict the knee resultant joint moment, with LRP applied to evaluate the impact of variations in kinematic features on model predictions. In previous model-based predictions, kinematic parameters were typically collected using inertial measurement units (IMU) or conventional optical motion capture systems to improve ecological validity.^43^ Considering the accuracy required during model training, this study employed a traditional optical motion capture system with higher precision.^44^ To reduce input dimensionality and model complexity, only sagittal plane joint angles were selected. This strategy helps focus on evaluating model performance while improving training stability and reducing the risk of overfitting under limited data conditions.

This study aims to construct a GNN-GRU-based deep learning model to estimate knee resultant joint moment in the supporting limb throughout the tennis serve, and to quantify the contributions of kinematic features related to knee joint injury using LRP. To further evaluate the model’s effectiveness, a standalone GRU model was constructed as a baseline for comparison. The ultimate goal is to provide a basis for assessing knee injury risk in tennis and to offer guidance for future injury prevention. It is hypothesized that the GNN-GRU model, compared to the GRU model alone, can more effectively integrate spatiotemporal features to accurately predict the knee resultant joint moment and successfully apply LRP to reveal reliable associations between sagittal plane joint angles of both lower limbs and the predicted moment, with the supporting knee joint expected to show a high contribution in the prediction task.

## Methods

**Figure 1.**
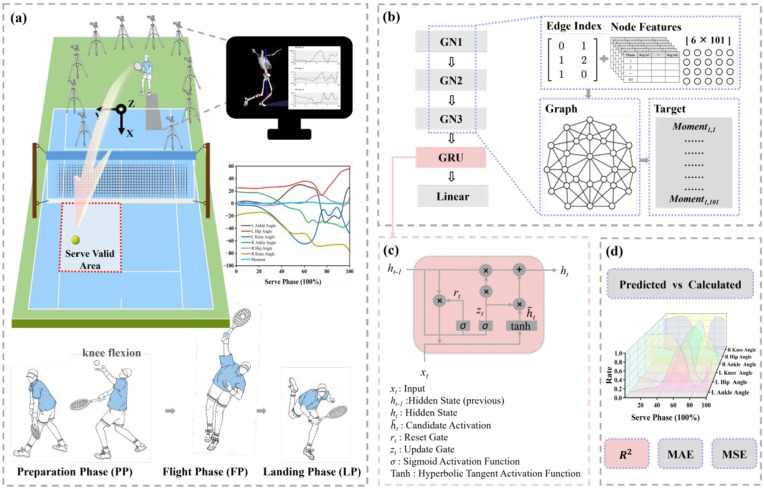
Research workflow. (a) Data collection and preprocessing. Kinematic and kinetic data were captured through a Vicon system with Kistler force plates, and processed using Visual3D. (b) GNN-GRU model training. A complete graph was constructed by combining node features and edge indices. Data were passed through three graph convolutional layers (GN1-GN3), then input into a GRU for temporal sequence modeling. The output was generated through a fully connected layer (Linear). (c) GRU architecture. (d) Model performance and prediction results.

In this study, lower limb biomechanical data during platform serves were collected from male tennis players. Raw data were preprocessed and categorized. Sagittal plane joint angles of both lower limbs and the knee resultant joint moment of the supporting leg were selected as model inputs. A GNN-GRU-based deep learning model was trained. LRP was then applied to identify kinematic features strongly associated with injury risk in the supporting knee joint (Figure 1).

### Participants

This study enrolled 30 male tennis players (age: 20.30 ± 1.66 years, height: 176.60 ± 2.74 cm, weight: 70.80 ± 3.89 kg, BMI: 22.71 ± 1.38 kg/m^2^, training experience: 9.20 ± 2.81 years). All participants volunteered and provided written informed consent. All players were fully familiarized with testing procedures prior to data collection. Inclusion criteria were defined as follows: (1) a minimum of 3 years of structured tennis training; (2) the left leg served as the supporting leg during the LP; (3) no lower limb or foot injury or illness in the past 6 months; (4) no other factors affecting the execution of a platform serve. This study was approved by the institutional ethics committee.

### Procedure

Lower limb biomechanics during the full platform serve were recorded using a 10-camera infrared Vicon motion capture system (Oxford Metrics Ltd, Oxford, UK) at 200 Hz and Kistler force plates (Kistler Instrumente AG, Winterthur, Switzerland) at 1000 Hz. After completing a dynamic warm-up (DWU),^45^ each athlete simulated a platform serve on the force plate. A serve was deemed valid when the ball reached the designated service area (Figure 1(a)), and a standardized 5-s rest interval was enforced between serves to ensure consistency.^46^

### Data processing

All data were imported in “*c3d” format and processed using Visual 3d (version 6, C-Motion, Inc., Germantown, MD, USA). The entire tennis serve was defined as a single motion unit and was divided into three phases: the PP, the FP, and the LP (Figure 1(a)).^47^ To account for bilateral coordination and the potential impact of the non-supporting leg on overall dynamic balance and load distribution, this study extracted joint angles of both lower limbs as input features. Considering the need to reduce input dimensionality and model complexity, and based on previous research^48^ indicating that sagittal plane moments dominate during landing, only sagittal plane kinematic parameters were used for modeling. The resultant joint moment of the supporting knee was defined as the model’ s output variable to reflect the overall mechanical load on the knee joint. The computation was carried out according to the following equation:

(1) $$\begin{array}{c} \mathrm{moment}=\sqrt{\mathrm{momen}{t_{x}}^{2}+\mathrm{momen}{t_{y}}^{2}+\mathrm{momen}{t_{z}}^{2}} \end{array}$$

Among them, moment_x_, moment_y_ and moment_z_ represent the joint moment components in the sagittal, frontal, and transverse planes, respectively. All motion signals were filtered using a fourth-order low-pass filter with a cutoff frequency of 10 Hz.^49^ Each participant completed 12 trials, yielding 360 valid serves in total. The dataset was partitioned into training (288 samples), validation (36 samples) and test sets (36 samples) using a cross-validation algorithm, with a ratio of 8:1:1.^43^ All time-series data were normalized to 101 points and exported as CSV files, yielding a 6 × 101 matrix for the input features and a 1 × 101 matrix for the resultant moment.

### Graph neural networks and gate recurrent units

A GNN-GRU integrated model was constructed (Figure 1(b) and (c)), using Python (Python Software Foundation, Wilmington, DE, USA) with the PyTorch library (Meta Platforms Inc., Menlo Park, CA, USA) and torch-geometric (Stanford University, Stanford, CA, USA). The model input was a 6 × 101 feature matrix, where each column represented a node and each row corresponded to a specific feature dimension for that node. One serve cycle was represented as a graph consisting of 101 nodes, each with six feature dimensions. Since edge features were not explicitly assigned, the edge attribute matrix was initialized as an all-ones matrix.^50^ A fully connected graph was constructed based on the uncontrolled manifold (UCM) theory,^51^ which suggests that motor tasks involve structured joint coordination. Therefore, each node was connected to all others to capture potential global dependencies.^52^

During model training, graph-structured data were first passed into graph convolutional layers, where spatial feature fusion was achieved by aggregating information from each target node and its neighbors. Three graph convolutional layers were used, each consisting of 64 neurons with ReLU activation. A GRU layer consisting of 64 units was subsequently applied to extract dynamic features across the temporal dimension (Figure 1(c)). The final output was produced through a fully connected layer. Model training used the Adam optimizer at a learning rate of 0.001 for 100 epochs. All model hyperparameters were optimized based on the results obtained through particle swarm optimization (PSO).^53^ To enhance interpretability, LRP was employed to quantify the contribution of each input variable to the predicted knee resultant joint moment.^41^ Model performance was evaluated by computing the MAE, MSE and R^2^ between predicted and actual values in the test set.^35^

### Statistics

After normality testing, the differences in MAE, MSE and *R*^2^ between the GNN-GRU model and the standalone GRU model were compared using SPSS 27.0.1 (IBM Corp, Armonk, NY, USA). To comprehensively analyze the temporal variation in the knee resultant joint moment, a paired-sample t-test was performed using the SPM1D toolbox in MATLAB (R2024a, The MathWorks, Natick, MA, USA) to compare the predicted and actual values generated by the GNN-GRU model. A significance threshold of 0.05 was applied.

## Results

The prediction performance for the six sagittal plane joint angles was evaluated using MAE, MSE, and *R*^2^ (Table 1). The GNN-GRU model showed significantly lower MAE and MSE, and significantly higher *R*^2^ compared to the GRU model (*p* < 0.05). The GNN-GRU model showed good performance across the three metrics, with the exception of the left ankle, where *R*^2^ was less than 0.8.

**Table 1.** Performance metrics for kinematic features predicted by the GNN-GRU and GRU models (n = 360).

Features	GNN-GRU			GRU			*p*	*d*
	MAE	MSE	R^2^	MAE	MSE	R^2^		
L ankle angle	0.209 (0.047)	0.080 (0.028)	0.799 (0.024)	0.339 (0.009)	0.214 (0.011)	0.424 (0.015)	0.000^a^	3.842^a^
							0.000^b^	6.299^b^
							0.000^c^	18.738^c^
L knee angle	0.204 (0.094)	0.077 (0.056)	0.828 (0.101)	0.440 (0.014)	0.324 (0.015)	0.127 (0.024)	0.000^a^	3.512^a^
							0.000^b^	6.025^b^
							0.000^c^	9.550^c^
L hip angle	0.202 (0.061)	0.074 (0.039)	0.820 (0.063)	0.314 (0.010)	0.194 (0.011)	0.477 (0.010)	0.000^a^	2.562^a^
							0.000^b^	4.188^b^
							0.000^c^	7.604^c^
R ankle angle	0.215 (0.096)	0.090 (0.069)	0.810 (0.096)	0.490 (0.009)	0.366 (0.016)	0.114 (0.081)	0.000^a^	4.033^a^
							0.000^b^	5.511^b^
							0.000^c^	8.962^c^
R knee angle	0.206 (0.055)	0.082 (0.029)	0.805 (0.014)	0.455 (0.012)	0.334 (0.018)	0.199 (0.022)	0.000^a^	6.255^a^
							0.000^b^	10.441^b^
							0.000^c^	38.288^c^
R hip angle	0.198 (0.071)	0.078 (0.037)	0.819 (0.037)	0.401 (0.012)	0.287 (0.012)	0.226 (0.018)	0.000^a^	3.987^a^
							0.000^b^	7.599^b^
							0.000^c^	20.382^c^

Data are presented as mean (SD). MAE denotes mean absolute error, MSE denotes mean squared error, and R^2^ denotes the coefficient of determination. d denotes Cohen’ s d, representing the effect size of the difference.

^a,b,c^Denote comparisons of MAE, MSE, and R^2^ between the two models, respectively.

**Figure 2.**
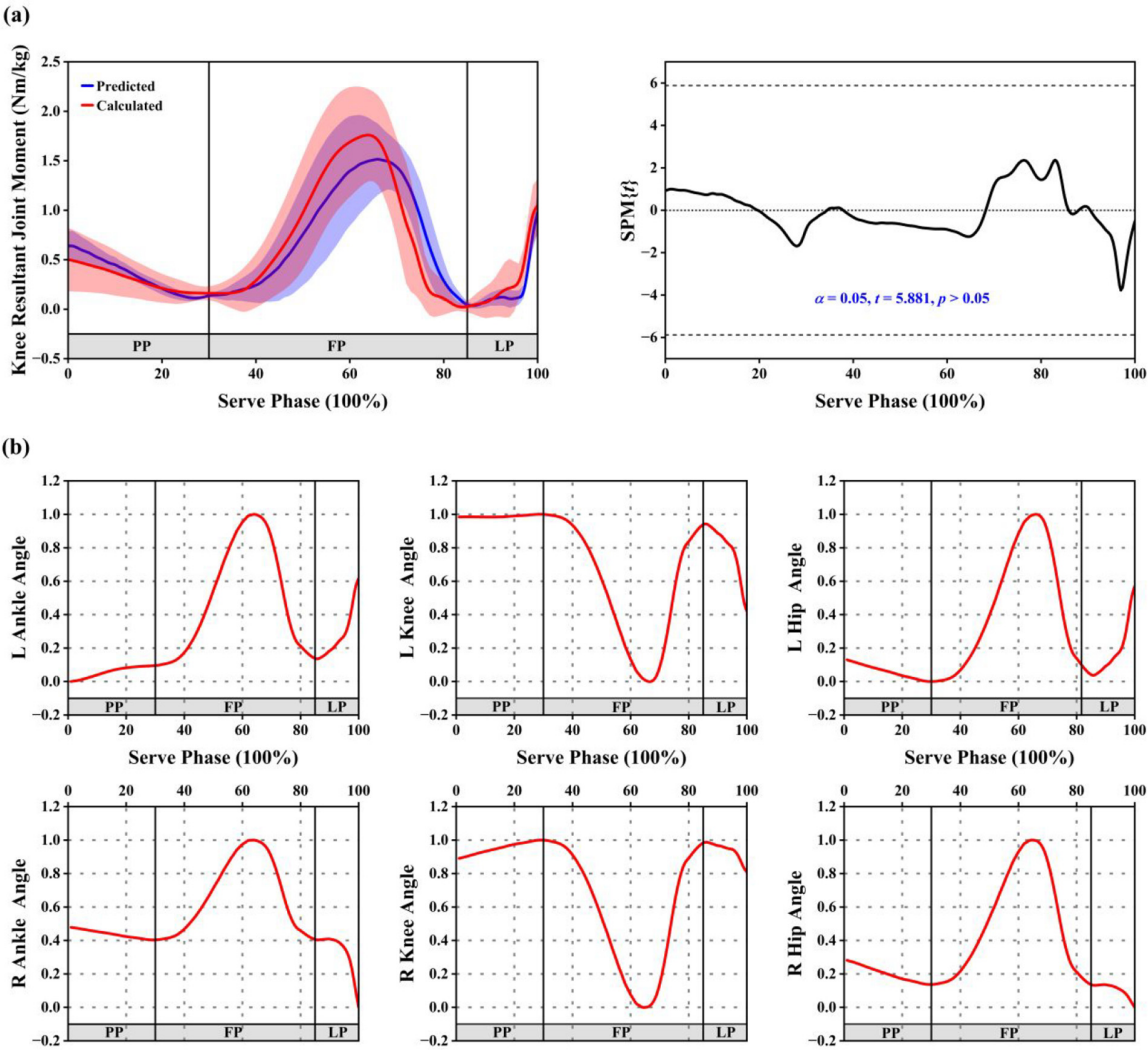
(a) SPM analysis comparing the knee resultant joint moment predicted by the GNN-GRU model with the actual computed values. (b) Temporal variation in the contributions of six input features determined by LRP. Contribution values were normalized to a range between 0 and 1. PP, FP, and LP represent the Preparation Phase, Flight Phase, and Landing Phase, respectively.

SPM results showed no statistically significant difference between the measured and predicted knee resultant joint moment values (*p* > 0.05; Figure 2(a)). During the PP, the knee resultant joint moment of the supporting leg gradually decreased. In the FP, it increased to a peak and then declined. In the LP, it gradually increased.

LRP was applied to identify the temporal dynamics of six input features’ contributions throughout the tennis serve (Figure 2(b)). Contribution scores ranged from 0 to 1, with 0 representing no effect on the dependent variable (the knee resultant joint moment of the supporting leg), and 1 representing maximum influence. During the PP, the contributions of the left ankle and right knee gradually increased, the left knee remained stable and close to 1, while the left hip, right ankle, and right hip gradually decreased. In the FP, the ankle and hip joints of both lower limbs showed a rise in contribution scores approaching 1, followed by a gradual decline, while the contributions of both knees decreased from near 1 to 0 and then increased again to near 1. In the LP, the contributions of the left ankle and left hip gradually increased. The right ankle and right hip initially remained stable but then decreased to near 0. The contributions of both knees declined from near 1.

## Discussion

This study implemented a GNN-GRU model combined with LRP to predict knee resultant joint moment of the supporting leg during the tennis serve using sagittal plane joint angles of both lower limbs as input. The results indicated that the GNN-GRU model, compared to the GRU model alone, more accurately predicted the knee resultant joint moment of the supporting leg and, through LRP, revealed the temporal variation in the influence of six kinematic variables on the predicted values. The model exhibited good overall performance.

### Model performance and prediction results

Compared to the standalone GRU model, the GNN-GRU model achieved superior prediction performance in terms of MAE, MSE, and *R*^2^. This improvement may be attributed to the GNN’ s ability to capture spatial dependencies among joints,^54^ which enhances the temporal modeling capability of GRU. Such spatiotemporal modeling is particularly beneficial for complex coordinated movements like the tennis serve. The GNN-GRU model developed in the current study exhibited strong predictive capacity, with the left knee achieving the highest *R*^2^ and relatively low MSE, reflecting strong predictive accuracy and explanatory power. This may be attributed to the substantial sagittal-plane flexion-extension activity of the supporting knee, which contributes directly to energy generation and transmission during serving actions.^2,55^ This process subjects the knee joint to high flexion-extension demands, increasing joint load and injury susceptibility. The model effectively captured the functional contribution of the right knee and hip as components of the lower limb kinetic chain. The left ankle showed slightly lower *R*^2^, while the right ankle exhibited the highest MAE with greater prediction variability, possibly due to individual differences in serving patterns among players.^56^ As the terminal segment of the lower limb kinetic chain, the ankle undertakes complex dynamic tasks during the tennis serve, being especially susceptible to variation arising from individual technical styles and landing strategies.^57^ This high degree of movement individuality increases data variability and may limit the model’s fitting capacity.

The results showed no significant difference between predicted and actual values, which suggests that the GNN-GRU model with simplified input features achieved high accuracy and reliability in predicting the resultant joint moment. During the PP, the moment of supporting knee gradually decreased, which may reflect a state of pre-activation in preparation for subsequent force generation. During the FP, the knee resultant joint moment first increased to a peak and then declined. In this phase, athletes must actively exert force during take-off to control movement stability and direction, while gradually unloading joint load during the aerial phase. The large moment fluctuation results in high instantaneous loading on the knee, elevating the risk of injury.^10^ However, this lower-limb–driven force generation is an essential technical element of the serve, critical for pre-impact energy storage and upward transfer.^2^ While preserving technical integrity, coordinated training may help reduce joint stress and injury risk. During the LP, the knee resultant joint moment increased again. At this stage, ground reaction force rises rapidly as the lower limbs touch down, requiring the knee muscles to absorb the impact.^58^ Inadequate control of this buffering process may lead to stress accumulation in knee structures and increase the risk of chronic injury.^59^

### Feature contribution rates

Following model validation, LRP was applied to quantify the contributions of six kinematic features. During the PP, both knees showed maximal contribution toward the end of the stage, indicating that sagittal plane knee angles played a dominant role in the prediction. This pattern aligns with task-specific demands, as athletes enter a phase of knee flexion at the end of PP,^47^ to generate greater racket speed through knee bending.^2^ Although knee loading increases during this motion, the injury risk remains lower than in the LP due to its active, performance-driven nature.

During the FP, ankle contribution peaked at the midpoint (64%) before declining, reflecting its role in propulsion via rapid plantarflexion.^57^ As the serve transitioned, both ankle and hip contributions decreased, while the knees gradually became the primary load-bearing joints. This shift corresponds to the body’s preparation for landing, where knee flexion plays a key role in absorbing impact forces and stabilizing motion.^58^

During the LP, the contribution of the left ankle gradually increased, suggesting a stronger role in knee load modulation and a possible association with injury risk. The ankle can assist in absorbing ground reaction forces through plantarflexion to aid in impact attenuation,^60^ but the rising contribution also suggests increased involvement in knee load regulation. Fong et al.^46^ similarly reported that limited ankle flexion-extension might contribute to a heightened likelihood of ACL injury. The contribution of the left hip also showed a gradual increase during the LP, indicating that hip flexion-extension played a contributory role in the occurrence of injury. Under normal conditions, the hip stabilizes pelvic and trunk posture, helping to distribute ground reaction forces while alleviating loading on the knee joint.^61^ Impaired hip motion can result in increased knee valgus and adduction moment, thereby raising injury risk.^62^ Leppänen et al.^63^ also identified a significant association between smaller hip flexion angles and a greater probability of ACL injury. In contrast, the contributions of the right, non-supporting ankle and hip showed a declining trend during this phase, suggesting a reduced role in impact absorption. At the beginning of the LP, both knees exhibited contribution values close to 1, which then slightly declined but remained at a relatively high level. This pattern indicates a strong association between knee flexion-extension and variations in the knee resultant joint moment. Inadequate flexion control before landing buffering is completed may lead to abnormal joint loading and increased injury risk.^64^ Targeted training to enhance knee flexion-extension control may help reduce landing-related injury risk.

The LRP visualization clearly illustrated the contributions of different lower limb joint features to the knee resultant joint moment of the supporting leg across the serve phases. In particular, the high contribution of knee flexion-extension during the LP suggests a strong association with knee loading and injury risk. Meanwhile, other joint kinematic features also showed certain levels of relevance to the knee resultant joint moment, reflecting the coordinated regulation among joints in the lower limb. During the tennis serve, the lower limb joints function as an integrated, dynamically regulated kinetic chain.^65^ Regular training should emphasize the coordinated development of the entire lower limb kinetic chain, with emphasis on key joints while also ensuring that the synergistic roles of other joints are not overlooked. Especially during landing buffering, sagittal plane coordination of lower limb joints plays a critical role in dispersing ground reaction forces,^66^ which helps mitigate injury risk. Therefore, enhancing the coordination of the whole kinetic chain should be considered a key objective for both injury prevention and performance optimization.^67^ Moreover, the difference in joint contributions between the supporting and non-supporting legs may reflect the inherent functional differentiation in unilateral actions such as the tennis serve. It has been reported that during the landing phase of the serve, the supporting leg plays a primary role in impulse absorption.^47^ The findings of this study are, to some extent, consistent with these biomechanical patterns.

Understanding the underlying mechanisms of the human musculoskeletal system is essential for biomechanical research on injury prevention and functional restoration.^9^ As machine learning techniques have advanced, numerous studies have concentrated on extracting human movement features and evaluating injury risk using data from video or inertial sensors.^38,43^ However, limited research has applied machine learning to infer joint moments based on kinematic input, and these attempts still exhibit notable prediction errors.^68,69^ In the study by Johnson and Ballard,^68^ the best-performing model achieved an RMSE slightly below 60 Nm. This study employed an LRP-based GNN-GRU model to assess the relative contributions of kinematic features associated with supporting leg knee injury to the prediction output. Using only sagittal plane joint angles of both lower limbs as input, the model accurately predicted the knee resultant joint moment during the tennis serve. The use of single-plane kinematic features simplifies data acquisition and processing. In future practical training and competition, this method could potentially be combined with existing approaches that estimate joint angles from images or videos,^38^ theoretically reducing dependence on complex measurement equipment and aiding in injury risk management. However, this study has certain limitations. The model relied solely on sagittal plane features, potentially overlooking contributions from movements in other planes to knee loading. Future research may incorporate multi-plane features to enhance prediction comprehensiveness. Additionally, laboratory-based experiments may not fully capture real-world tennis variability, and video-based prediction applications still require further validation. In the future, data collected from actual training or competition scenarios, including match videos, could be incorporated to enhance model robustness and verify its generalizability in practical applications. Moreover, this study evaluated only a single model without comparison to other modeling approaches. Future research could introduce multiple models for comparative analysis to better assess and optimize performance.

## Conclusions

This study employed a GNN-GRU model with LRP to accurately predict the knee resultant joint moment of the supporting leg during the tennis serve, using sagittal plane joint angles of both lower limbs as input. Compared to the baseline GRU model, the GNN-GRU model achieved superior performance across all evaluation metrics, demonstrating high goodness-of-fit, with no significant difference between predicted and actual values. These results highlight the spatiotemporal variations in joint coordination across different serve phases, indicating that different joint groups contribute to the modulation of knee joint moments at specific time points. This may provide a reference for developing targeted monitoring tools or preventive strategies in tennis-specific training. Future studies should validate these findings in real-world sports environments and explore the potential integration with wearable devices or vision-based recognition systems to support on-court biomechanical monitoring.

## References

[1] Girard O Micallef JP Millet GP. Lower-limb activity during the power serve in tennis: effects of performance level. Med Sci Sports Exerc 2005; 37: 1021–1029. 15947729

[2] Hornestam JF Souza TR Magalhães FA , et al. The effects of knee flexion on tennis serve performance of intermediate level tennis players. Sensors 2021; 21: 5254. 34450697 10.3390/s21165254 PMC8398391

[3] Pluim BM Staal JB Windler GE , et al. Tennis injuries: occurrence, aetiology, and prevention. Br J Sports Med 2006; 40: 415–423. 16632572 10.1136/bjsm.2005.023184 PMC2577485

[4] Dines JS Bedi A Williams PN , et al. Tennis injuries: epidemiology, pathophysiology, and treatment. J Am Acad Orthop Surg 2015; 23: 181–189. 25667400 10.5435/JAAOS-D-13-00148

[5] Pan J Sun D Li F , et al. The effects of skill level on lower-limb injury risk during the serve landing phase in male tennis players. Appl Sci 2025; 15: 2681.

[6] Dakic JG Smith B Gosling CM , et al. Musculoskeletal injury profiles in professional Women’s Tennis Association players. Br J Sports Med 2018; 52: 723–729. 29074474 10.1136/bjsports-2017-097865

[7] Renström AF. Knee pain in tennis players. Clin Sports Med 1995; 14: 163–175. 7712548

[8] Hame SL Oakes DA Markolf KL. Injury to the anterior cruciate ligament during alpine skiing: a biomechanical analysis of tibial torque and knee flexion angle. Am J Sports Med 2002; 30: 537–540. 12130408 10.1177/03635465020300041301

[9] Hewett TE Myer GD Ford KR , et al. Biomechanical measures of neuromuscular control and Valgus loading of the knee predict anterior cruciate ligament injury risk in female athletes: a prospective study. Am J Sports Med 2005; 33: 492–501. 15722287 10.1177/0363546504269591

[10] Sigward SM Powers CM. Loading characteristics of females exhibiting excessive valgus moments during cutting. Clin Biomech 2007; 22: 827–833. 10.1016/j.clinbiomech.2007.04.003 17531364

[11] Challis JH Kerwin DG. Quantification of the uncertainties in resultant joint moments computed in a dynamic activity. J Sports Sci 1996; 14: 219–231. 8809714 10.1080/02640419608727706

[12] Kellis E. The effects of fatigue on the resultant joint moment, agonist and antagonist electromyographic activity at different angles during dynamic knee extension efforts. J Electromyogr Kinesiol 1999; 9: 191–199. 10328414 10.1016/s1050-6411(98)00032-7

[13] Stefanyshyn DJ Lee J-S Park S-K. The influence of soccer cleat design on resultant joint moments. Footwear Sci 2010; 2: 13–19.

[14] DeMorat G Weinhold P Blackburn T , et al. Aggressive quadriceps loading can induce noncontact anterior cruciate ligament injury. Am J Sports Med 2004; 32: 477–483. 14977677 10.1177/0363546503258928

[15] Kristianslund E Krosshaug T van den Bogert AJ. Artefacts in measuring joint moments may lead to incorrect clinical conclusions: the nexus between science (biomechanics) and sports injury prevention! Br J Sports Med 2013; 47: 470–473. 22872681 10.1136/bjsports-2012-091199

[16] Buchanan TS Lloyd DG Manal K , et al. Estimation of muscle forces and joint moments using a forward-inverse dynamics model. Med Sci Sports Exerc 2005; 37: 1911–1916. 16286861 10.1249/01.mss.0000176684.24008.6f

[17] Versteyhe M De Vroey H Debrouwere F , et al. A novel method to estimate the full knee joint kinematics using low cost IMU sensors for easy to implement low cost diagnostics. Sensors 2020; 20: 1683. 32197330 10.3390/s20061683 PMC7147475

[18] Kanko RM Laende E Selbie WS , et al. Inter-session repeatability of markerless motion capture gait kinematics. J Biomech 2021; 121: 110422. 33873117 10.1016/j.jbiomech.2021.110422

[19] Xiang L Gu Y Deng K , et al. Integrating personalized shape prediction, biomechanical modeling, and wearables for bone stress prediction in runners. Digit Med 2025; 8: 276–312. 10.1038/s41746-025-01677-0 PMC12075602 40360731

[20] Zaroug A Lai DTH Mudie K , et al. Lower limb kinematics trajectory prediction using long short-term memory neural networks. Front Bioeng Biotechnol 2020; 8: 362. 32457881 10.3389/fbioe.2020.00362 PMC7227385

[21] Moghadam SM Yeung T Choisne J. A comparison of machine learning models’ accuracy in predicting lower-limb joints’ kinematics, kinetics, and muscle forces from wearable sensors. Sci Rep 2023; 13: 5046. 36977706 10.1038/s41598-023-31906-z PMC10049990

[22] Wang D Li S Song Q , et al. Predicting vertical ground reaction force in rearfoot running: a wavelet neural network model and factor loading. J Sports Sci 2023; 41: 955–963. 37634140 10.1080/02640414.2023.2251767

[23] Yamane T Kimura M Morita M. Application of nine-axis accelerometer-based recognition of daily activities in clinical examination. Phys Act Heal 2024; 8: 29–46.

[24] Yamane T Kimura M Morita M. Impact of sensor-axis combinations on machine learning accuracy for human activity recognition using accelerometer data in clinical settings. Phys Act Heal 2025; 9: 95–109. DOI: 10.5334/paah.441

[25] Whiteside D Cant O Connolly M , et al. Monitoring hitting load in tennis using inertial sensors and machine learning. Int J Sports Physiol Perform 2017; 12: 1212–1217. 28182523 10.1123/ijspp.2016-0683

[26] Li J Zhang X Yang G. The biomechanical analysis on the tennis batting angle selection under deep learning. IEEE Access 2023; 11: 97758–97768.

[27] Liu S. Tennis players’ hitting action recognition method based on multimodal data. Int J Biom 2024; 16: 317–336.

[28] Ganser A Hollaus B Stabinger S. Classification of tennis shots with a neural network approach. Sensors 2021; 21: 5703. 34502593 10.3390/s21175703 PMC8433919

[29] Du Y Xia Y Wang L , et al. The influence of psychological change factors of tennis training strategy using optimized recurrent neural network and artificial intelligence. Heliyon 2024; 10: e33273. 39027517 10.1016/j.heliyon.2024.e33273 PMC11255447

[30] Ding G Plummer A Georgilas I. Deep learning with an attention mechanism for continuous biomechanical motion estimation across varied activities. Front Bioeng Biotechnol 2022; 10: 1021505. 36324889 10.3389/fbioe.2022.1021505 PMC9618651

[31] Xiang L Wang A Gu Y , et al. Recent machine learning progress in lower limb running biomechanics with wearable technology: a systematic review. Front Neurorobot 2022; 16: 913052. 35721274 10.3389/fnbot.2022.913052 PMC9201717

[32] Mirzavand Borujeni S Arras L Srinivasan V , et al. Explainable sequence-to-sequence GRU neural network for pollution forecasting. Sci Rep 2023; 13: 9940. 37336995 10.1038/s41598-023-35963-2 PMC10279754

[33] Gao Z Xiang L Fekete G , et al. A data-driven approach for fatigue detection during running using pedobarographic measurements. Appl Bionics Biomech 2023; 2023: 1–11. 10.1155/2023/7022513 PMC10547577 37794856

[34] Xu S Rao H Peng H , et al. Attention-based multilevel co-occurrence graph convolutional LSTM for 3-D action recognition. IEEE Internet Things J 2021; 8: 15990–16001.

[35] Song X Deng L Wang H , et al. Deep learning-based time series forecasting. Artif Intell Rev 2024; 58: 23.

[36] Li ZL Zhang GW Yu J , et al. Dynamic graph structure learning for multivariate time series forecasting. Pattern Recognit 2023; 138: 109423.

[37] Zhu J Ye Z Ren M , et al. Transformative skeletal motion analysis: optimization of exercise training and injury prevention through graph neural networks. Front Neurosci 2024; 18: 1353257. 38606310 10.3389/fnins.2024.1353257 PMC11008465

[38] Wu B Chen W Liu D , et al. MyoStep: feature-based GNN model for estimating knee joint angles by fusing signals from sEMG and IMU. IEEE Sens J 2025; 25: 17750–17760.

[39] Yang C Jin P Chen Y. Leveraging graph neural networks and gate recurrent units for accurate and transparent prediction of baseball pitching speed. Sci Rep 2025; 15: 7745. 40044722 10.1038/s41598-025-88284-x PMC11882905

[40] Guidotti R Monreale A Ruggieri S , et al. A survey of methods for explaining black box models. ACM Comput Surv 2019; 51: 1–42.

[41] Ullah I Rios A Gala V , et al. Explaining deep learning models for tabular data using layer-wise relevance propagation. Appl Sci 2021; 12: 136.

[42] Horst F Lapuschkin S Samek W , et al. Explaining the unique nature of individual gait patterns with deep learning. Sci Rep 2019; 9: 2391. 30787319 10.1038/s41598-019-38748-8 PMC6382912

[43] Halilaj E Rajagopal A Fiterau M , et al. Machine learning in human movement biomechanics: best practices, common pitfalls, and new opportunities. J Biomech 2018; 81: 1–11. 30279002 10.1016/j.jbiomech.2018.09.009 PMC6879187

[44] Salisu S Ruhaiyem NIR Eisa TAE , et al. Motion capture technologies for ergonomics: a systematic literature review. Diagnostics 2023; 13: 2593. 37568956 10.3390/diagnostics13152593 PMC10416907

[45] Ayala F Moreno-Pérez V Vera-Garcia FJ , et al. Acute and time-course effects of traditional and dynamic warm-up routines in young elite junior tennis players. PLoS One 2016; 11: e0152790. 27071014 10.1371/journal.pone.0152790 PMC4829215

[46] Fong C-M Blackburn JT Norcross MF , et al. Ankle-dorsiflexion range of motion and landing biomechanics. J Athl Train 2011; 46: 5–10. 21214345 10.4085/1062-6050-46.1.5 PMC3017488

[47] Kovacs M Ellenbecker T. An 8-stage model for evaluating the tennis serve: implications for performance enhancement and injury prevention. Sports Health Multidiscip Approach 2011; 3: 504–513. 10.1177/1941738111414175 PMC3445225 23016050

[48] Devita P Skelly WA. Effect of landing stiffness on joint kinetics and energetics in the lower extremity. Med Sci Sports Exerc 1992; 24: 108–115. 1548984

[49] Olmedilla Zafra A Prieto Andreu JM Blas Redondo A . Relaciones entre estrés psicosocial y lesiones deportivas en tenistas. Univ Psychol 2010; 10: 909–922.

[50] Yi Y Lu X Gao S , et al. Graph classification via discriminative edge feature learning. Pattern Recognit 2023; 143: 109799.

[51] Scholz JP Schöner G. The uncontrolled manifold concept: identifying control variables for a functional task. Exp Brain Res 1999; 126: 289–306. 10382616 10.1007/s002210050738

[52] Maldonado G Bailly F Souères P , et al. On the coordination of highly dynamic human movements: an extension of the uncontrolled manifold approach applied to precision jump in parkour. Sci Rep 2018; 8: 12219. 30111843 10.1038/s41598-018-30681-6 PMC6093881

[53] Gad AG. Particle swarm optimization algorithm and its applications: a systematic review. Arch Comput Methods Eng 2022; 29: 2531–2561.

[54] Chen Y You J He J , et al. SP-GNN: Learning structure and position information from graphs. Neural Netw 2023; 161: 505–514. 36805265 10.1016/j.neunet.2023.01.051

[55] Chen Y Wang T Zhao Y , et al. Kinematic differences between female national and provincial athletes in the tennis serve. PeerJ 2024; 12: e18410. 39494283 10.7717/peerj.18410 PMC11531263

[56] Elliott BC. Biomechanics of the serve in tennis. A biomedical perspective. Sports Med 1988; 6: 285–294. 3064237 10.2165/00007256-198806050-00004

[57] Mourtzios C Athanailidis I Arvanitidou V , et al. Ankle and knee joint kinematics differ between flat, slice and topspin serves in young tennis players. Eur J Sport Sci 2022; 1: 16–22.

[58] Lopes TJA Simic M Myer GD , et al. The effects of injury prevention programs on the biomechanics of landing tasks: a systematic review with meta-analysis. Am J Sports Med 2018; 46: 1492–1499. 28759729 10.1177/0363546517716930 PMC6604048

[59] Paterno MV Schmitt LC Ford KR , et al. Biomechanical measures during landing and postural stability predict second anterior cruciate ligament injury after anterior cruciate ligament reconstruction and return to sport. Am J Sports Med 2010; 38: 1968–1978. 20702858 10.1177/0363546510376053 PMC4920967

[60] Schmitz RJ Kulas AS Perrin DH , et al. Sex differences in lower extremity biomechanics during single leg landings. Clin Biomech 2007; 22: 681–688. 10.1016/j.clinbiomech.2007.03.001 17499896

[61] Hashemi J Breighner R Chandrashekar N , et al. Hip extension, knee flexion paradox: a new mechanism for non-contact ACL injury. J Biomech 2011; 44: 577–585. 21144520 10.1016/j.jbiomech.2010.11.013

[62] Pollard CD Sigward SM Powers CM. Limited hip and knee flexion during landing is associated with increased frontal plane knee motion and moments. Clin Biomech 2010; 25: 142–146. 10.1016/j.clinbiomech.2009.10.005 PMC2815098 19913961

[63] Leppänen M Pasanen K Krosshaug T , et al. Sagittal plane hip, knee, and ankle biomechanics and the risk of anterior cruciate ligament injury: a prospective study. Orthop J Sports Med 2017; 5: 2325967117745487. 29318174 10.1177/2325967117745487 PMC5753918

[64] Leppänen M Pasanen K Kujala UM , et al. Stiff landings are associated with increased ACL injury risk in young female basketball and floorball players: Response. Am J Sports Med 2017; 45: 386–393. 27637264 10.1177/0363546516665810

[65] Kovalchik SA Reid M. Comparing matchplay characteristics and physical demands of junior and professional tennis athletes in the era of big data. Am J Sports Sci Med 2017; 16: 489–497. PMC5721178 29238248

[66] Blackburn JT Padua DA. Influence of trunk flexion on hip and knee joint kinematics during a controlled drop landing. Clin Biomech 2008; 23: 313–319. 10.1016/j.clinbiomech.2007.10.003 18037546

[67] Zhou Z Li S Yang L , et al. Inter-segmental coordination of the swimming start among paralympic swimmers: a comparative study between S9, S10, and S12 swimmers. Appl Sci 2023; 13: 9097.

[68] Johnson L Ballard D. Efficient codes for inverse dynamics during walking. Proc AAAI Conf Artif Intell 2014; 28: 343–349.

[69] Ozates ME Karabulut D Salami F , et al. Machine learning-based prediction of joint moments based on kinematics in patients with cerebral palsy. J Biomech 2023; 155: 111668. 37276682 10.1016/j.jbiomech.2023.111668

